# MiRNA sequencing of Embryonic Myogenesis in Chengkou Mountain Chicken

**DOI:** 10.1186/s12864-022-08795-z

**Published:** 2022-08-10

**Authors:** Jun’an Shi, Wendong Li, Anfang Liu, Lingtong Ren, Pusen Zhang, Ting Jiang, Yuqing Han, Lingbin Liu

**Affiliations:** College of Animal Science and Technology, Chongqing Key Laboratory of Herbivore Science, Southwest University, Beibei, Chongqing, 400700 China

**Keywords:** MicroRNA, WGCNA, Chengkou mountain chicken, Embryonic Myogenesis

## Abstract

**Background:**

Skeletal muscle tissue is among the largest organ systems in mammals, essential for survival and movement. Embryonic muscle development determines the quantity and quality of muscles after the birth of an individual. MicroRNAs (miRNAs) are a significant class of non-coding RNAs that bind to the 3’UTR region of mRNA to regulate gene function. Total RNA was extracted from the leg muscles of chicken embryos in different developmental stages of Chengkou Mountain Chicken and used to generate 171,407,341 clean small RNA reads. Target prediction, GO, and KEGG enrichment analyses determined the significantly enriched genes and pathways. Differential analysis determined the significantly different miRNAs between chicken embryo leg muscles at different developmental stages. Meanwhile, the weighted correlation network analysis (WGCNA) identified key modules in different developmental stages, and the hub miRNAs were screened following the KME value.

**Results:**

The clean reads contained 2047 miRNAs, including 721 existing miRNAs, 1059 known miRNAs, and 267 novel miRNAs. Many genes and pathways related to muscle development were identified, including *ERBB4*, *MEF2C*, *FZD4*, the Wnt, Notch, and MAPK signaling pathways. The WGCNA established the greenyellow module and gga-miR-130b-5p for E12, magenta module and gga-miR-1643-5p for E16, purple module and gga-miR-12218-5p for E19, cyan module and gga-miR-132b-5p for E21.

**Conclusion:**

These results lay a foundation for further research on the molecular regulatory mechanism of embryonic muscle development in Chengkou mountain chicken and provide a reference for other poultry and livestock muscle development studies.

**Supplementary Information:**

The online version contains supplementary material available at 10.1186/s12864-022-08795-z.

## Background

Skeletal muscles develop from the mesoderm during the embryonic development of vertebrates such as birds and mammals [1]. Poultry has two kinds of fine muscle fiber; red and white. The leg muscle mainly consists of red muscle fiber. Poultry muscle is an important protein source for humans. The main edible parts are the pectoral and leg muscles, and all are skeletal muscles. Skeletal muscle is an important vertebrate tissue accounting for 40% of body weight [2] and mainly functions in movement, postural support, breathing, and thermogenesis [3].

Moreover, skeletal muscle myogenesis is a complex biological process affected by various regulators [4–7]. During embryogenesis, the skeletal muscle forms in the vertebrate limb from progenitor cells originating in the somites [8]. The process takes four stages to develop into mature muscle fibers. In the first stage, the mesenchymal stem cells from the mesoderm undergo terminal differentiation to form mononuclear myoblasts. The second stage involves the fusion of the mononuclear myoblasts to form a fusiform multinucleated myotube. Then, the third stage involves further differentiation of muscle tubes into muscle fibers. The last stage involves the growth and eventual maturation of muscle fibers [9, 10]. Thus, microRNAs can be employed to study the four stages of skeletal muscle development.

MicroRNAs are a class of endogenous small non-coding RNAs, approximately 19–22 nucleotides long, playing various important regulatory roles in cells, such as regulating post-transcriptional gene expression in plants and animals. Approximately 70% of mammalian miRNAs are located in transcription units (TUs) [11], but most miRNAs are located in introns. Each miRNA can target multiple genes, and several miRNAs can regulate the same gene. MiRNA maturation involves several processes [12], including 1) Primary miRNA formation by RNA polymerase II; 2) pre-miRNA generation by the nuclear RNase III enzyme, Drosha and its cofactor DGCR8 cleavaged. Next, 3) the pre-miRNA form miR/miR* duplex (an siRNA-like duplex) via the RNase III enzyme, Dicer; and lastly, 4) the mature single-stranded miRNA from the duplex is incorpoeated into the RNA-induced silencing complex (RISC). MiRNA functions by binding to AGO protein to form RISC, which then binds to the 3 ‘UTR region of the functional gene [13].

This study used transcriptomics, the study of gene expression at the RNA level, to analyze the skeletal muscles of Chengkou Mountain Chicken. The transcriptomics technology, also known as RNA-Seq, is an important method for studying cell phenotype and function. The transcriptome is the sum of transcription products of all genes in a cell, including mRNA, rRNA, tRNA, and non-coding RNA at a specific state or physiological condition of an organism. Therefore, a major feature of the transcriptome is its spatiotemporal specificity. The recent development of the next-generation high-throughput sequencing technology has tremendously updated the transcriptome sequencing technology. New technologies such as single-cell transcriptome sequencing and spatial transcriptome sequencing are discovered, has extended transcriptome research extends to the translatome and structurome [14], greatly enriching scientific output.

China is a vast country with diverse terrain and species. Chongqing, located in southwest China is the hub city of southwest China and the economic center of the upper reaches of the Yangtze River. The Chongqing climate is within the northern subtropical mountain area, characterized by many mountains and rivers. The climate is mild, with abundant rainfall, sufficient sunshine, and four distinct seasons, making it very suitable for the growth and breeding of animals and plants. The area has abundant high-quality local livestock and poultry varieties. The Chengkou mountain chicken is a local poultry variety with excellent product features, including resistance to coarse feeding, delicious meat, strong resistance, and high nutritional value. However, it has similar challenges (slow growth rate and low meat yield) as other local breeds [15]. Therefore, studying and clarifying the biological mechanism of muscle development is necessary for improving the production performance of Chengkou mountain chicken and retaining its advantages.

This study analyzed Chengkou mountain chicken to explore the superiority of local chicken species in heritage performance by investigating four-stage chicken leg muscles: 12-day (E12), 16-day (E16), 19-day (E19), and 21-day (E21) embryos. Small-RNA sequencing unraveled differentially expressed miRNAs involved in embryo development. The study further characterized the differentially expressed miRNA in muscle development and established the enrichment functions and structure of miRNAs.

## Results

### Overview of small-RNA sequencing

**Fig. 1:**
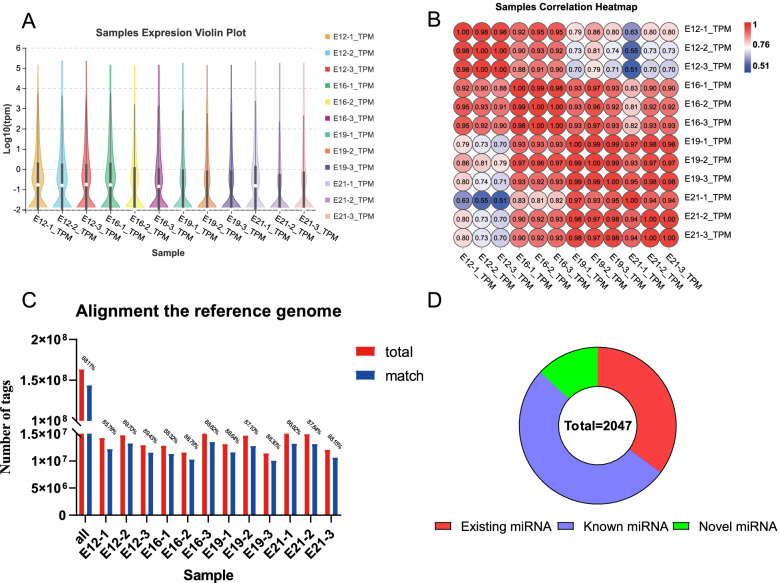
Overview of sequencing data. **A** Samples miRNA expression violin plot; **B** Samples correlation heatmap; **C** Align ment the reference genome; **D** miRNA species distribution

We constructed 12 cDNA libraries (E12-1, E12-2, E12-3, E16-1, E16-2, E16-3, E19-1, E19-2, E19-3, E21-1, E21-2, and E21-3) from embryo leg muscle to obtain complete miRNA transcripts of the chicken embryo. A total of 171,407,341 clean reads were generated from 12 cDNA libraries after dropping low-quality reads. Thus, reads containing over one low-quality base or unknown nucleotides (N); without 3’ adaptors; containing 5’ adaptors; containing 3’ and 5’ adaptors but no small RNA fragment between them; containing polyA in small RNA fragments < 18 nt were removed. The remaining high-quality reads of each duplicate were approximately 99%, and the proportion of clean tags was > 94% (Table 1). Transcripts per million(TPM) showed that miRNAs had different expressions (Fig. 1A), and samples correlation heatmaps showed high reproducibility between all samples (Fig. 1B). The length distribution of small RNA sequences was approximately 22 bp, consistent with conventional animal samples (Fig. S1). Nearly 5% of the tags aligned to non-coding RNAs (including rRNA, scRNA, snRNA, snoRNA, and tRNA) based on the GenBank (Release 209.0, Table S1) and the Rfam (version 11.0, Table S2) databases. The additional 95% of the tags were used for follow-up analysis. Moreover, over 85% of the transcripts had a high genome match (Fig. 1C). The reference area statistics showed consistent proportions of sense and antisense tags in the exon and intron regions (Fig. S2). The repeat alignment results are shown in Table S3.

**Table 1:** Sequencing data quality control

id	Clean reads	High quality	3’adapter null	Insert null	5’adapter contaminants	Smaller than 18nt	PolyA	Low cutoff	Clean tags
E12-1	15,182,867 (100%)	15,032,507 (99.0097%)	11,259 (0.0749%)	27,699 (0.1843%)	7291 (0.0485%)	353,353 (2.3506%)	365 (0.0024%)	374,772 (2.4931%)	14,257,768 (94.8462%)
E12-2	15,486,862 (100%)	15,337,952 (99.0385%)	13,217 (0.0862%)	25,360 (0.1653%)	5054 (0.0330%)	251,801 (1.6417%)	273 (0.0018%)	246,696 (1.6084%)	14,795,551 (96.4637%)
E12-3	13,546,502 (100%)	13,426,336 (99.1129%)	8751 (0.0652%)	18,045 (0.1344%)	4443 (0.0331%)	203,557 (1.5161%)	231 (0.0017%)	230,529 (1.7170%)	12,960,780 (96.5325%)
E16-1	13,440,948 (100%)	13,298,384 (98.9393%)	21,028 (0.1581%)	16,664 (0.1253%)	4725 (0.0355%)	200,716 (1.5093%)	500 (0.0038%)	215,551 (1.6209%)	12,839,200 (96.5471%)
E16-2	12,182,274 (100%)	12,082,685 (99.1825%)	6212 (0.0514%)	15,911 (0.1317%)	5505 (0.0456%)	250,947 (2.0769%)	302 (0.0025%)	191,460 (1.5846%)	11,612,348 (96.1073%)
E16-3	15,876,789 (100%)	15,709,214 (98.9445%)	5765 (0.0367%)	20,027 (0.1275%)	4689 (0.0298%)	209,133 (1.3313%)	560 (0.0036%)	230,806 (1.4692%)	15,238,234 (97.0019%)
E19-1	13,754,500 (100%)	13,645,117 (99.2047%)	5805 (0.0425%)	19,585 (0.1435%)	3795 (0.0278%)	248,176 (1.8188%)	460 (0.0034%)	232,089 (1.7009%)	13,135,207 (96.2631%)
E19-2	15,351,792 (100%)	15,198,195 (98.9995%)	6913 (0.0455%)	20,152 (0.1326%)	4133 (0.0272%)	247,101 (1.6259%)	469 (0.0031%)	211,815 (1.3937%)	14,707,612 (96.7721%)
E19-3	12,025,634 (100%)	11,935,295 (99.2488%)	4363 (0.0366%)	16,022 (0.1342%)	3712 (0.0311%)	290,870 (2.4371%)	264 (0.0022%)	173,365 (1.4525%)	11,446,699 (95.9063%)
E21-1	16,230,582 (100%)	15,989,901 (98.5171%)	50,361 (0.3150%)	27,090 (0.1694%)	4495 (0.0281%)	331,264 (2.0717%)	504 (0.0032%)	341,409 (2.1352%)	15,234,778 (95.2775%)
E21-2	15,518,503 (100%)	15,363,022 (98.9981%)	6122 (0.0398%)	13,592 (0.0885%)	4329 (0.0282%)	168,750 (1.0984%)	182 (0.0012%)	177,280 (1.1539%)	14,992,767 (97.5900%)
E21-3	12,810,088 (100%)	12,699,534 (99.1370%)	3490 (0.0275%)	19,648 (0.1547%)	6717 (0.0529%)	378,662 (2.9817%)	240 (0.0019%)	208,206 (1.6395%)	12,082,571 (95.1418%)

### Identification of microRNA

The tag abundance identified as miRNA was > 70%, and the miRNAs identified in this study were divided into three categories. (1) Existing miRNA; the miRNA obtained by comparing existing miRNAs of Chicken in the miRBase database. (2) Known miRNA; the miRNA obtained by comparing miRNA of other species in the miRBase database. (3) Novel miRNA; the new miRNA obtained by hairpin structure prediction based on comparing small RNA with reference sequences. About 7% of the miRNA was base-edited for each sample (Table S5), and 1,059 known miRNA were identified (Table S6). The first nucleotide bias within the existing miRNA tag sequences was U (Fig. S3), and the first nucleotide bias with known miRNA sequences was A and U (Fig. S4). In summary, 2047 miRNAs were identified by classifying 266,267 tags, including 721 existing miRNAs (Table S4), 1059 known miRNAs, and 267 novel miRNAs (Table S7, Fig. 1D). Fig. S5 shows the tag annotations for different samples.

### MicroRNA different expression analysis

**Fig. 2:**
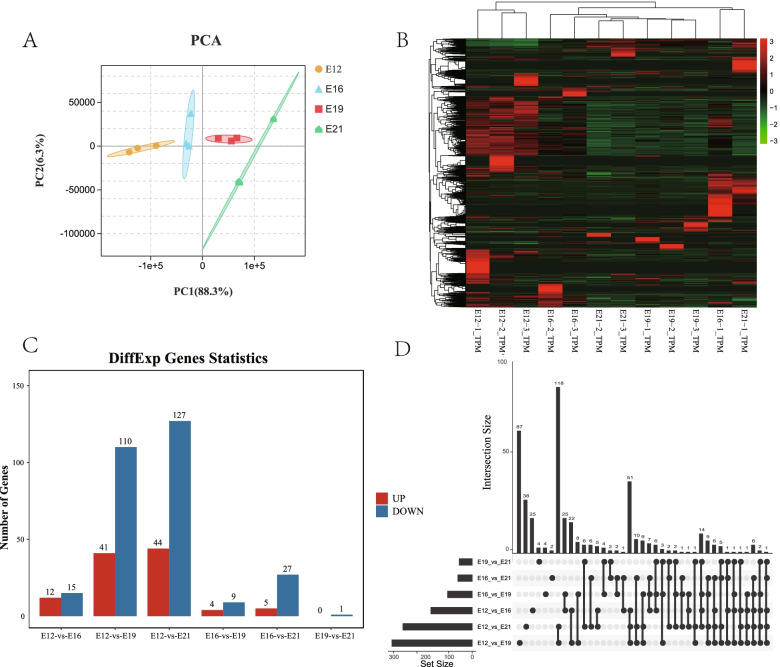
Differential expression of lncRNAs analysis. **A** The PCA distribution of 12 samples; **B** Differential miRNA cluster analysis; **C** Differential miRNA statistics at different time points; **D** The upset plot of miRNA expression at different time point

The PCA analysis of all the miRNA with < 1 TPM revealed 12 samples divided into four groups by time point (Fig. 2A). Samples E19 and E21 were very close, probably because both are in late embryonic development, indicating the reliability of the sequence data. Meanwhile, the cluster analysis showed that most miRNAs are expressed in the early stage embryos, indicating the importance of miRNA in early embryonic development (Fig. 2B). The edgeR software identified 196 differentially expressed miRNAs, including 27 in E12_vs_E16, 151 in E12_vs_E19, 171 in E12_vs_E21, 13 in E16_vs_E19, 32 in E16_vs_E21, and 1 in E19_vs_E21 (Fig. 2C). We performed an Upset plot on miRNAs from different stages to identify key miRNAs in muscle development. The results showed that the larger the time span, the more differentially expressed genes (Fig. 2D).

### Weighted correlation network analysis (WGCNA) of miRNAs

**Fig. 3:**
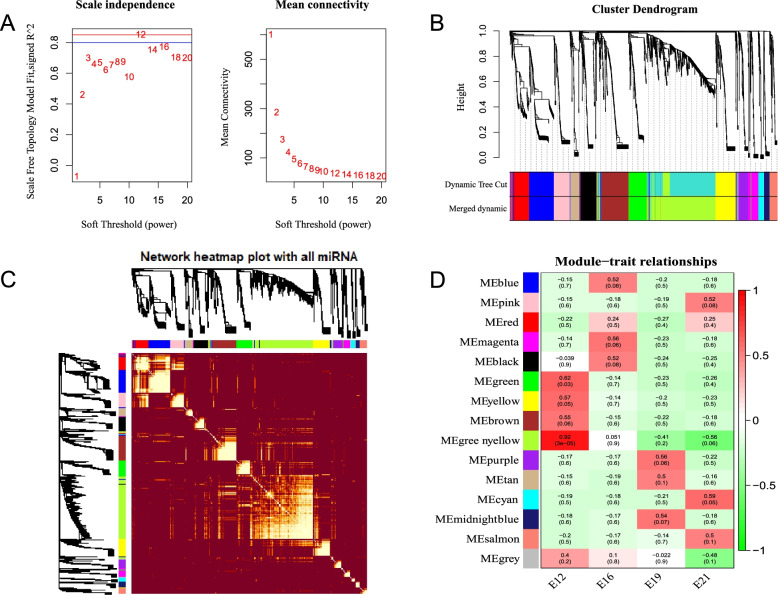
Weighted gene co-expression network analysis of miRNAs. **A** The Power value curve; **B** Module eigenvalue clustering; **C** Module gene correlation analysis; **D** Correlation analysis of traits

The “WGCNA” R package [16] identified the key module miRNAs associated with and their regulatory roles in the different stages of embryonic muscle development. Essentially, 12 soft thresholds were used to ensure that the module conforms to scale-free distribution (Fig. 3A). Therefore, 15 modules (excluding unclassified miRNAs) were identified in the different module colors (Fig. 3B). The brighter the color of the intersection between the row and column, the closer the gene connection between the corresponding row and column. The Pearson correlation was stronger (Fig. 3C). Association analysis revealed significant correlations between E12 and greenyellow (*r* = 0.92), E16 and magenta (*r* = 0.58), E19 and purple (*r* = 0.58), and E21 and cyan (*r* = 0.59) (Fig. 3D). High KME (eigengene connectivity) values indicated hub genes with the most connections. The top3 miRNAs with the highest KME in each module were chosen as hub miRNAs of the corresponding modules (Table 2).

**Table 2:** The expression of hub miRNAs

module	id	E12-1	E12-2	E12-3	E16-1	E16-2	E16-3	E19-1	E19-2	E19-3	E21-1	E21-2	E21-3
greenyellow	gga-miR-130b-5p	1460.273	1406.085	1336.597	687.8573	767.8346	722.779	429.3586	477.7194	434.7701	400.8186	312.7624	372.9574
	gga-miR-1552-5p	156.7062	146.5911	142.0809	62.7668	81.752	66.0258	37.1165	36.2199	39.0731	21.4229	22.2508	28.5149
	gga-miR-363-5p	123.4279	104.4432	103.9048	61.4366	56.0239	64.7641	34.0094	40.7198	35.4188	23.5952	20.1648	23.1741
magenta	gga-miR-1643-5p	0.01	0.01	0.01	0.01	0.01	0.1402	0.01	0.01	0.01	0.01	0.01	0.01
	gga-miR-1709	0.01	0.01	0.01	0.01	0.01	0.1402	0.01	0.01	0.01	0.01	0.01	0.01
	gga-miR-1796	0.01	0.01	0.01	0.01	0.01	0.1402	0.01	0.01	0.01	0.01	0.01	0.01
purple	gga-miR-12218-5p	0.01	0.01	0.01	0.01	0.01	0.01	0.01	0.01	0.1874	0.01	0.01	0.01
	gga-miR-6667-5p	0.01	0.01	0.01	0.01	0.01	0.01	0.01	0.01	0.1874	0.01	0.01	0.01
	miR-2984-y	0.01	0.01	0.01	0.01	0.01	0.01	0.01	0.01	0.2811	0.01	0.01	0.01
cyan	gga-miR-132b-5p	0.01	0.01	0.01	0.01	0.01	0.01	0.01	0.01	0.01	0.01	0.3477	0.01
	gga-miR-132b-3p	0.01	0.01	0.01	0.01	0.01	0.01	0.01	0.01	0.01	0.01	0.1391	0.01
	gga-miR-1592	0.01	0.01	0.01	0.01	0.01	0.01	0.01	0.01	0.01	0.01	0.1391	0.01

### Functional analysis of miRNAs and co-expressed genes

**Fig. 4:**
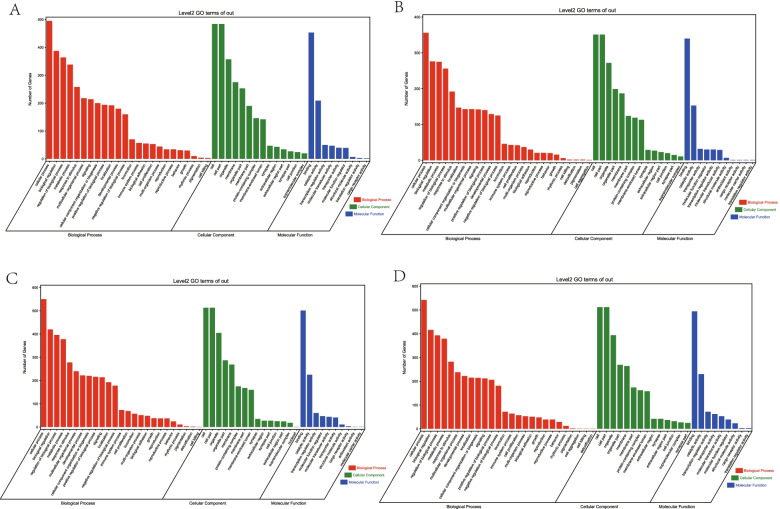
The enriched GO terms of the DE miRNAs. **A** E12: gga-miR-130b-5p target genes; **B** E16: gga-miR-1643-5p target genes; **C** E19: gga-miR-12218-5p target genes; **D** E21: gga-miR-132b-5p target genes

The main function of miRNA is to bind mRNA regulate the expression of target genes. Top1 hub miRNA in four modules with different muscle development time points were selected for subsequent analysis of enriched miRNAs. Subsequently, target genes were predicted (total predicted miRNA target genes were listed in Table S9). The GO enrichment analysis of the miRNAs that target genes showed several muscle development-related GO terms in E12, including the regulation of muscle tissue development, muscle organ development, and muscle tissue development (Fig. 4A). In E16, the regulation of muscle cell differentiation and the regulation of vascular associated smooth muscle cell migration were enriched (Fig. 4B). Nonetheless, the regulation of vascular smooth muscle cell differentiation and the vascular smooth muscle cell differentiation were enriched in E19 (Fig. 4C). The skeletal muscle satellite cell differentiation and skeletal muscle cell differentiation were enriched in E21 (Fig. 4D).

**Fig. 5:**
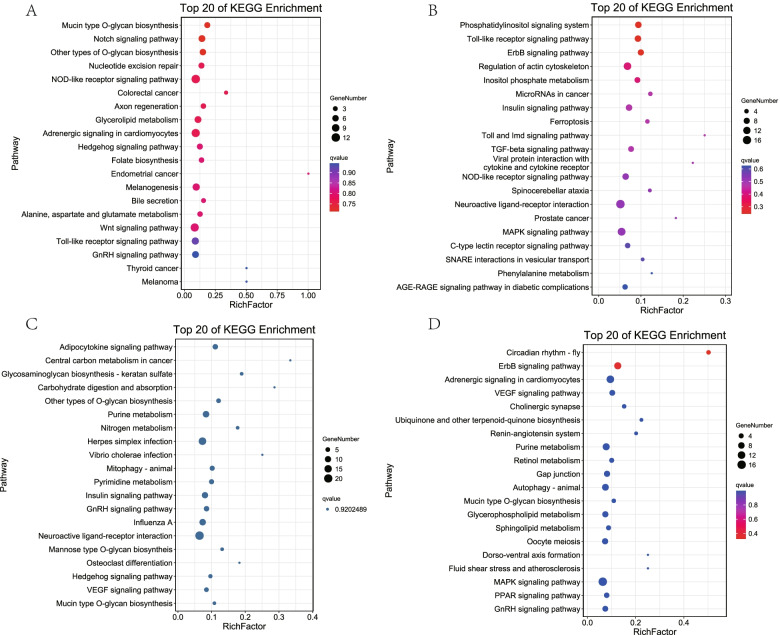
The enriched KEGG pathways of the DE miRNAs. **A** E12: gga-miR-130b-5p target genes; **B** E16: gga-miR-1643-5p target genes; **C** E19: gga-miR-12218-5p target genes; **D** E21: gga-miR-132b-5p target genes

A Kyoto Encyclopedia of Genes and Genomes (KEGG, http://www.genome.jp/kegg/) revealed the miRNA enriched pathways. Consequently, the mucin-type O-glycan biosynthesis and Notch signaling pathways were the most significantly enriched in E12 (Fig. 5A). The phosphatidylinositol signaling system, toll-like, and ErbB signaling pathways were the most significantly enriched in E16 (Fig. 5B). In E19, the adipocytokine signaling pathway was the most significantly enriched (Fig. 5C), while the circadian rhythm-fly and the ErbB signaling pathways were the most significantly enriched in E21 (Fig. 5D). Meanwhile, some star signaling pathways were significantly enriched during different developmental periods, such as the Wnt (E12), GnRH (E12, E19, E21), MAPK (E16, E21), and PPAR (E21) signaling pathways.

### Co-expression network establishment

**Fig. 6:**
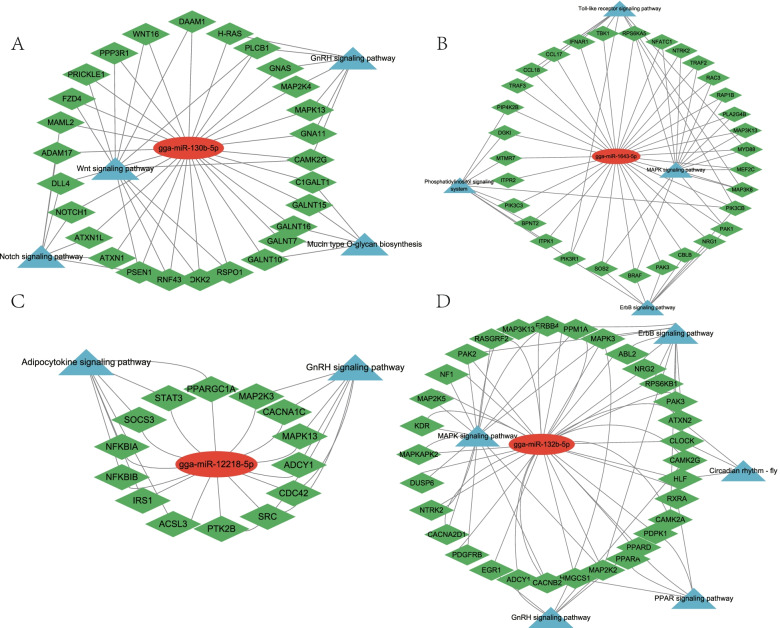
Interaction network of miRNA, mRNA and key signal pathway. **A** E12: gga-miR-130b-5p target genes and pathways; **B** E16: gga-miR-1643-5p target genes and pathways; **C** E19: gga-miR-12218-5p target genes and pathways; **D** E21: gga-miR-132b-5p target genes and pathways

Based on previous analyses, we focused on a few pathways related to embryo muscle development combined with a previous mRNA study [17]. The key miRNA-mRNA-pathway regulatory networks for different embryo development stages were built via Cytoscape 3.9.1. Thus, the Wnt signaling pathway was involved in development at E12, and the key genes included *DAAM1*, *WNT16*, *PPP3R1*, *PRICKLE1*, *FZD4*, *PSEN1*, *PLCB1*, *CAMK2G*, and *RSPO1* (Fig. 6A). MAPK signaling was the most significant pathway for gga-miR-1643-5p at E16, and the important genes included *MAP3K13*, *SOS2*, *BRAF*, *PAK1*, *RAP1B*, *RAC3*, *TRAF2*, *MYD88*, *NTRK2*, *NFATC1*, *RPS6KA5*, *MAP3K8*, and *MEF2C* (Fig. 6B). Similarly, the most important pathway for E19 and E21 were adipocytokine and MAPK signaling pathways, respectively. The key genes included *SOCS3*, *ACSL3*, *IRS1*, *STAT3*, *NFKBIA*, *PPARGC1A*, and *PPM1A* for the adipocytokine signaling pathway. In contrast, *ERBB4*, *MAP3K13*, *MAPK3*, *RASGRF2*, *PAK2*, *MAP2K2*, *NF1*, *MAP2K5*, *KDR*, *MAPKAPK2*, *DUSP6*, *NTRK2*, *CACNA2D1*, *PDGFRB*, *CACNB2* were key in the MAPK signaling pathway (Fig. 6C-D).

### Validation of candidate miRNAs and miRNA-mRNA relationship

**Fig. 7:**
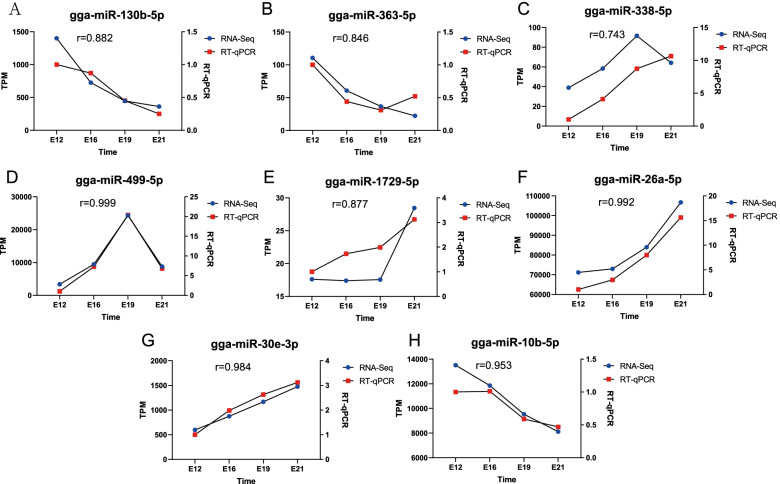
The validation of candidate miRNAs. **A** gga-miR-130b-5p; **B** gga-miR-363-5p; **C** gga-miR-338-5p; **D** gga-miR-499-5p; **E** gga-miR-1729-5p; **F** gga-miR-26a-5p; **G** gga-miR-30e-3p; **H** gga-miR-10b-5p. Blue means RT-qPCR, red means RNA-Seq and r means correlation coefficient. U6 was used as the reference gene for RT-qPCR, RNA-Seq relative expression was represent by TPM

**Fig. 8:**
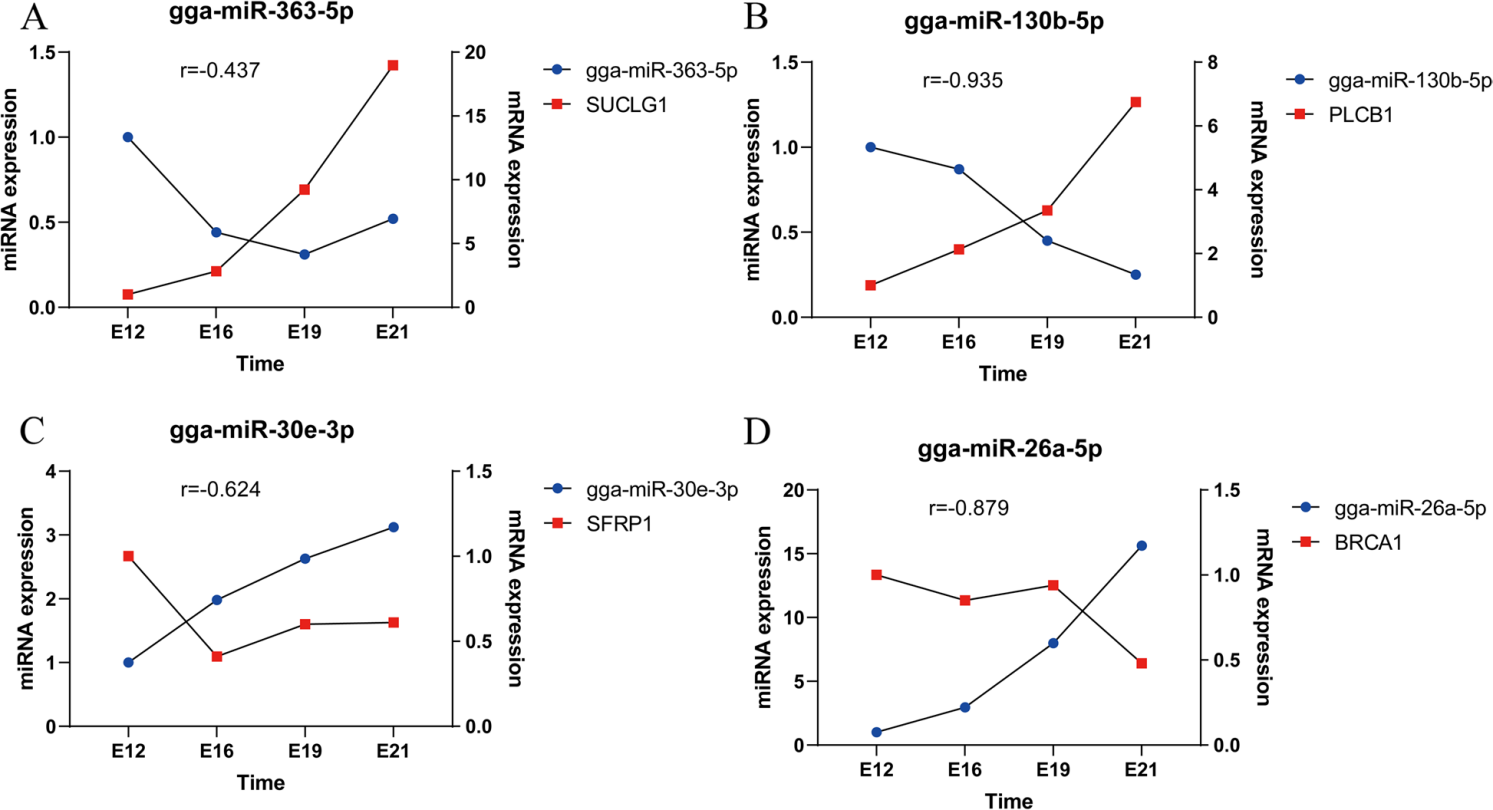
The validation of correlation miRNA-mRNA. **A** gga-miR-363-5p – SUCLG1; **B** gga-miR-130b-5p – PLCB1; **C** gga-miR-30e-3p – SFRP1; **D** gga-miR-26a-5p – BRCA1

The miRNAs with high KME values and high expression in the key modules corresponding to different developmental stages of Chengkou Mountain Chicken embryos were selected for RT-qPCR. They included gga-miR-130b-5p, gga-miR-363-5p, gga-miR-338-5p, gga-miR-499-5p, gga-miR-1729-5p, gga-miR-26a-5p, gga-miR-30e-3p, and gga-miR-10b-5p. The RT-qPCR results and small RNA-Seq results were highly correlated (Fig. 7), confirming the accuracy of the sequencing results. Four pairs of miRNA-mRNA were randomly selected for RT-qPCR based on candidate miRNAs and target gene prediction results, and found that although there was a significant negative correlation of miRNA-mRNA expression relationship (Fig. 8).

## Discussion

Muscle development, growth, and regeneration occur throughout the life cycle of vertebrates. Myogenesis occurs in four consecutive, time-distinct but overlapping stages in amniotes, including embryo, fetus, neonate, and adult [18]. Fetal and neonatal myogenesis is key for muscle growth and maturation. Adult myogenesis is necessary for postpartum growth and repairing damaged muscles [19]. Primary and secondary fibers are produced during poultry embryonic and fetal development; after that, the number of myofibers remains stable [20] except during damage repair.

The small RNA sequence detection range is 18-30nt endogenous RNA, including miRNA, siRNA, and piRNA. However, the main objective of this study was to detect miRNAs related to muscle development of the chicken embryo to promote the genetic improvement of Chengkou mountain chickens. Therefore, 2047 miRNAs were detected by small RNA sequencing of the embryonic leg muscle of Chengkou Mountain chickens at different developmental time points. The differential analysis identified 196 differentially expressed miRNAs, indicating the significance of the 196 miRNAs in the muscle development of the chicken embryo.

A WGCNA systematic biological method described the patterns of gene association among the different samples and identified highly covariant gene sets, candidate biomarker genes, or therapeutic targets for use in medical and biological fields. WGCNA revealed the TERT^high^-specific miR-17–92 cluster can targets biological processes enriched in the TERT^low^ cancer in a pan-cancer analysis [21]. Elsewhere, RNA sequencing and WGCNA of cord blood samples from fetal growth-restricted cases and controls combined with maternal peripheral blood quantification revealed miR-42-5p and miR-1306-3p as potential fetal growth restrictors [22]. In calves, WGCNA identified bta-miR-145 and bta-miR-199a-3p as important hub miRNAs regulating rumen development, immune system, and protein digestion [23]. This technique also established that *DYNLL2* and its target miR-148-3p are important regulators of chicken myogenesis [24]. In this study, the WGCNA technique revealed key modules and hub genes related to leg muscles of embryos at four different development stages. Thus, many interesting genes for muscle development-related biological process and signaling pathways were identified through GO and KEGG hub gene target analysis. The WGCNA analysis identified 537 miRNAs in the greenyellow module, highly correlated (*r* = 0.92) with E12, 78 miRNAs in the magenta module for E16, 78 miRNAs in the purple module for E19, and 49 miRNAs in the cyan module for E21. These results account for a quarter of the total muscle miRNA, indicating that the progression of muscle development in the embryo is extremely important during this period. The results are consistent with an earlier study of E12 to E21, the period of rapid maturity of muscle fiber during the embryonic development of Chengkou mountain chicken [17].

Previous studies have shown that miRNAs are involved in muscle development; miR-222a and miR-126-5p significantly reduced the *CPEB3* and *FGFR3* mRNA levels in chicken embryo fibroblasts [25], emphasizing the significance of miRNA-target interactions in embryonic muscle regulation. A luciferase reporter gene assay showed that miR-1 targets chicken *ACVR2B* UTR directly, but network analysis predicted that *ACVR2B* targets gga-miR-101, gga-miR-1a and gga-miR-499 [26]. Although gga-miRNA-454-3p does not affect primary myoblast differentiation, it inhibits differentiation by targeting the myotube-associated protein *SBF2* [27]. A study focused on the chicken skeletal muscle indicated that miR-29b-1-5p inhibits the proliferation of chicken primary myoblasts and promotes the differentiation of myoblasts via an effective target gene, *ANKRD9* [28]. Meanwhile, miR-29b-1-5p and miR-133a-5p are sponges for *circFGFR2* in skeletal muscle proliferation and differentiation [29]. MiR-133 was earlier established as a miRNA specifically expressed in skeletal muscles [30]. High-throughput sequencing revealed novel_miR_158, novel_miR_144, novel_miR_291, and miR-205a as crucial miRNAs for skeletal muscle development in Bian chicken, suggesting their vital function in chicken growth [31].

The proliferation and differentiation of skeletal muscle satellite cells are crucial in skeletal muscle development, especially during repair after muscle injury. MiR-21-5p targets *KLF3* and regulates skeletal muscle satellite cell proliferation and differentiation [32]. MicroR-27b-3p regulates the proliferation and differentiation of chicken primary myoblasts by targeting *MSTN* [33]. Moreover, miRNA-214 regulates chicken myoblast proliferation and differentiation by targeting *TRMT61A* [34]. The miRNA-gene pairs gga-miR-499-5p/*SOX6* and gga-miR-196-5p/*CALM1* might affect muscle fiber performance using a miRNA-mRNA integrated analysis [35]. All these miRNAs, except novel_miRNA (naming rules were different), were detected in this sequencing data, and gga-miR-499-5p was the candidate hub miRNA within the purple module for E19, suggesting its role in chicken muscle development.

RT-qPCR results by randomly selecting miRNAs and their target genes showed that there was extremely significant correlation between some miRNAs and mRNA, however, there were some miRNAs and mRNA with no obvious correlation. It confirmed that the targeting relationship between miRNA and mRNA is not unique. Besides, gga-miR-130b-5p, gga-miR-1643-5p, gga-miR-12218-5p, and gga-miR-132b-5p were critical for muscle development at E12, E16, E19, and E21 embryo stages in Chengkou mountain chickens. Multiple genes related to muscle development were identified by predicting the targets of these miRNAs. *MEF2C*, a member of the myocyte enhancer factor 2 family of *MADS* (MCM1, agamous, deficiens, serum response factor), is an important regulator of cardiac myogenesis and right ventricular development. This gene is mainly expressed in cardiac precursor cells before linear cardiac tube formation in mice, and *MEF2C* mutation prevents right ventricle formation in mice [36].

Meanwhile, *MEF2C* probably synergizes with *MyoD* through amplification to establish skeletal muscle commitment during cardiac and skeletal myogenesis [37]. Activation of satellite cells regulates the repair of injured human skeletal muscles. However, after knocking out the *MEF2A*, *MEF2C*, and *MEF2D* genes, satellite cells only proliferated but failed to differentiate, showing the dependence of skeletal muscle regeneration on *MEF2* [38]. *ERBB4* (Erb-B2 receptor tyrosine kinase 4), a Tyr protein kinase family member and the epidermal growth factor receptor subfamily, regulates muscle differentiation [39]. The Janus kinase/signal transducer and activator of transcription (JAK/STAT) signaling cascade has also been identified as a key factor in myogenesis. Nonetheless, the *STAT3* isoform is critical for satellite cell migration and myogenic differentiation because it mediates the expression of muscle-specific myogenic factors [40]. The *SOCS* (Suppressor of Cytokine Signaling) family of proteins down-regulates *STAT* activation [41]. Other target genes such as *BRAF* [42], *DAAM1* [43], *FZD4* [44, 45], and *NF1* [46, 47] affect muscle development in the same or different ways.

GO, and KEGG enrichment results showed that some muscle development entries were significantly enriched, including the Wnt, ErbB, MAPK, and Notch signaling pathways. As previously predicted, *DAAM1*, *FZD4*, and *WNT16* constitute the Wnt signaling pathway that regulates the critical ability of muscles to break down and reorganize fibers during development. Wnt signaling is involved in muscle remodeling [48]. Consequently, correct activation of the Wnt signaling pathway is essential during the various steps of muscle formation [49]. Therefore, deficiency of Wnt signaling effectors during pregnancy leads to marked tissue damage and muscle dysplasia [50]. *ERBB4* represents the ErbB signaling pathway, whose inhibition leads to non-denervated skeletal muscle growth in mice, but activation causes an opposite outcome [51].

In this study, the predicted members of the MAPK signaling pathway were *PPM1A*, *NF1*, *MEF2C*, and *TRAF2*. Ras-MAPK signaling promotes neuroactivity-dependent differentiation of slow muscle fibers in vivo [52]. Besides, the p38 MAPK is activated during myoblast differentiation, and it also affects the activity of the *MEF2* family of transcription factors, suggesting that p38 may be involved in the myogenic program [53]. Early morphogenesis of skeletal muscles during chicken embryo development requires transient activation of the Notch signaling pathway to drive terminal differentiation of muscle progenitors [54]. Notch and NRG signaling antagonistically regulate the synthesis and degradation of the cardiomyocyte extracellular matrix in a mouse trabecular model, which is critical for the individualization and rearrangement growth of trabecular units [55]. Generally, the growth and development of organisms is a complex process, often regulated by several signaling pathways. Studies have shown that myogenic progenitor cell differentiation transitions from Notch to Wnt signaling. The temporal balance between Notch and Wnt signaling coordinates the precise progression of muscle precursor cells along the myogenic lineage pathway [56].

## Conclusions

This work constructed the miRNA sequencing library of Chengkou mountain chicken, generating 2047 miRNAs and 196 differentially expressed miRNAs. Key modules, hub miRNAs, and targets corresponding to different chicken embryo developmental stages were identified through WGCNA and functional enrichment analysis. GO and KEGG enrichment analysis of target genes revealed several significantly enriched signaling pathways during embryonic muscle development, including the Wnt, ErbB, MAPK, and Notch signaling pathways. This report is highly consistent with previous mRNA sequencing results [17]. Combining these reports can provide a more accurate molecular basis for exploring the embryonic muscle development of Chengkou Mountain Chicken and guide the genetic improvement of local breeds.

## Methods

### Chicken embryo incubation and tissue collection

This study used the Chengkou mountain chicken as the experimental animal. Chengkou mountain chicken breeding eggs were obtained from the Chongqing Xuanpeng Agricultural Development Co. Ltd Chongqing, China. The eggs were incubated at 37.8 °C and 55% humidity. Twelve chicken embryos were obtained from four time points (12, 16, 19, and 21 embryonic ages), with three replicates at each time point. The embryos were euthanized via cervical spine dislocations, and leg muscles were collected from the same sampling sites. The 12 samples were stored at -80 ℃ (wrapped in RNA protective solution (QIAGEN, Hilden, Germany)) for RNA extraction.

### cDNA library construction and sequencing

The Trizol reagent (Invitrogen, USA) was used to extract total RNA from chicken embryo leg muscles during the four stages, following the manufacturer’s protocol. The RNA molecules within 18–30 nt were enriched by polyacrylamide gel electrophoresis (PAGE). Nucleic acid tests and gel electrophoresis assessed total RNA quality and purity. rRNA was removed from the total RNA using the Ribo-Zero rRNA removal kit (Epicentre, USA). The Illumina HiSeq™ 2500 (Illumina, CA, USA) was used for sequencing at the GENE DENOVO Biotechnology co. LTD (Guangzhou, China). The original data were filtered as follows to ensure quality. Reads containing: (1) > 1low-qualityy base (Q-value ≤ 20) or unknown nucleotides (N), (2) without 3’ adaptors, (3) containing 5’ adaptors, (4) containing 3’ and 5’ adaptors but no small RNA fragment in between, (5) containing polyA in small RNA fragments and < 18 nt were excluded. The clean reads were compared with the GenBank and Rfam species databases using the Blastall tool. Meanwhile, the chicken genome short reads were aligned using the tool Bowtie. Reads within the databases were divided and compared to avoid no mismatches. Then, the reserved unmapped reads were used for subsequent transcriptome analysis. The TPM determined the sample expression. Sample repeatability was tested via principal component analysis (PCA).

### Identification of miRNAs

All clean tags were searched against the miRbase database (Release 22) to identify existing miRNAs and known miRNAs via alignment with other species. The novel miRNAs were identified according to their genome positions and hairpin structures as predicted by the Mireap_v0.2 software. The default parameters of the Mireap_v0.2 software were as follows: (1) 18nt minimal and (2) 26nt maximal miRNA sequence length. (3) Minimal, 20nt, and (4) maximal miRNA sequence length, 24nt. (5) Minimal depth of Drosha/Dicer cutting site, 3, (6) maximal copy number of miRNAs on reference, 20, and (7) maximal free energy allowed for a miRNA precursor, 18 kcal/mol. (8) Maximal space between miRNA and miRNA*, 35nt, (9) minimal space between miRNA and miRNA*, 14nt, and (10) maximal bulge between miRNA and miRNA*, 4nt. (11) Maximal asymmetry of miRNA/miRNA* duplex, 5nt, and (12) flank sequence length of miRNA precursor, 10nt. The tag annotation results were determined in this priority order: rRNA etc. > existing miRNA > existing miRNA edit > known miRNA > repeat > exon > novel miRNA > intron. The tags that were not annotated at any of the above molecules were recorded as unannotated.

### MiRNA expression analysis

The total miRNA consisted of existing miRNA, known miRNA, and novel miRNAs, based on their expression in each sample. The miRNA expression was calculated and normalized to TPM. In addition, the expression of existing miRNA, known miRNA, and novel miRNA was also analyzed individually. The edgeR tool revealed the significantly different miRNAs based on the *P* value < 0.05 and |log2FC|> 1 threshold. RNAhybrid (Version 2.1.2) + svm_light (Version 6.01), Miranda (Version 3.3a) and TargetScan (Version 7.0) were used to predict targets. The intersection of the results was more credible and chosen as predicted miRNA target genes.

### Function enrichment analysis

The miRNA-mRNA regulatory relationship was constructed to analyze the function of target genes and clarify the mechanism of miRNA involvement in chicken embryo muscle development. The Genes to GO term mapping database calculated the number of genes in each GO term and GO functional statistics [57]. A hypergeometric test identified the significantly enriched GO entries compared with the entire genome background. A hypergeometric test used the KEGG databases to identify significantly enriched pathways against the entire genome background [58]. The most important biochemical metabolic and signal transduction pathways were determined through enrichment analysis. The calculated *p*-values were subjected to FDR correction, and pathways with FDR ≤ 0.05 were considered statistically significant.

### Verification and statistical analysis

Herein, eight miRNAs and four mRNAs were used to verify the sequencing results and the relationship between miRNAs and mRNAs expression levels via RT-qPCR. The primers were designed by Primer Premier (Table S8). RNA reverse transcription and real-time fluorescence quantitative PCR were performed as previously described [17], U6 and ACTB were used as housekeeping genes for miRNA and mRNA, respectively. The relative miRNA and mRNA expression were calculated via the 2^−△△CT^ method [59], and data were expressed as mean ± standard deviation of the mean. Duncan’s Multiple Range Test was used for two-group comparisons in SPSS 23.0 (SPSS Inc., IL, USA). Graphics were plotted using GraphPad Prism 9 (GraphPad Software, CA, USA). *P* < 0.05 and *P* < 0.01 were considered statistically significant and extremely significant, respectively [60].

## Supplementary Information

**Additional file 1: Table S1.** Alignment non-coding RNA in GenBank. **Table S2.** Alignment non-coding RNA in Rfam. **Table S3.** Alignment the repeat area. **Table S4.** Number of identified miRNA and tag abundance statistics. **Table S5.** Base-edited for each sample. **Table S6.** Statistics for identifying the known miRNA. **Table S7.** Statistics of novel miRNA. **Table S8.** The primers for the RT-qPCR amplification. **Figure S1.** Sample tags length distribution. (1) E12-1; (2) E12-2; (3) E12-3; (4) E16-1; (5) E16-2; (6); E16-3; (7) E19-1; (8) E19-2; (9) E19-3; (10) E21-1; (11) E21-2; (12) E21-3. **Figure S2.** Comparison of reference area statistics. Sample reference ratios distribution (1) E12-1; (2) E12-2; (3) E12-3; (4) E16-1; (5) E16-2; (6); E16-3; (7) E19-1; (8) E19-2; (9) E19-3; (10) E21-1; (11) E21-2; (12) E21-3. Replace different components ratios with different colors. **Figure S3.** The first nucleotide bias distribution. Replace different nucleotides with different colors. **Figure S4.** The first nucleotide bias with known miRNAs. Replace different nucleotides with different colors. **Figure S5.** Tags annotation for all samples. (1) E12-1; (2) E12-2; (3) E12-3; (4) E16-1; (5) E16-2; (6); E16-3; (7) E19-1; (8) E19-2; (9) E19-3; (10) E21-1; (11) E21-2; (12) E21-3. Replace different components ratios with different colors.

**Additional file 2: Table S9.** The predicted miRNA target genes.

## Data Availability

The raw data has been submitted to the National Center for Biotechnology Information (NCBI) Sequence Read Archive (SRA), and the accession number is SRP290982.
