# Supplementary material for: MiRNA sequencing of Embryonic Myogenesis in Chengkou Mountain Chicken
Source: BMC Genomics. 2022 Aug 10;23:571. doi: 10.1186/s12864-022-08795-z (PMC9364561; doi:10.1186/s12864-022-08795-z)

Supplementary material

Table S1 Alignment non-coding RNA in GenBank

| sample | total | rRNA | scRNA | snRNA | snoRNA | tRNA |
| --- | --- | --- | --- | --- | --- | --- |
| all | 163303515 | 5594913(3.43%) | 157393(0.10%) | 106537(0.07%) | 132900(0.08%) | 205255(0.13%) |
| E12-1 | 14257768 | 798250(5.60%) | 25598(0.18%) | 18178(0.13%) | 16416(0.12%) | 25018(0.18%) |
| E12-2 | 14795551 | 770048(5.20%) | 20600(0.14%) | 16206(0.11%) | 17618(0.12%) | 17951(0.12%) |
| E12-3 | 12960780 | 423799(3.27%) | 13678(0.11%) | 14385(0.11%) | 14617(0.11%) | 13572(0.10%) |
| E16-1 | 12839200 | 211306(1.65%) | 8380(0.07%) | 4961(0.04%) | 6246(0.05%) | 16380(0.13%) |
| E16-2 | 11612348 | 332993(2.87%) | 9093(0.08%) | 8039(0.07%) | 9501(0.08%) | 19027(0.16%) |
| E16-3 | 15238234 | 333699(2.19%) | 9561(0.06%) | 8639(0.06%) | 13553(0.09%) | 16968(0.11%) |
| E19-1 | 13135207 | 526016(4.00%) | 12304(0.09%) | 6871(0.05%) | 9393(0.07%) | 15713(0.12%) |
| E19-2 | 14707612 | 485370(3.30%) | 15478(0.11%) | 6386(0.04%) | 11245(0.08%) | 17729(0.12%) |
| E19-3 | 11446699 | 286586(2.50%) | 8146(0.07%) | 4642(0.04%) | 8178(0.07%) | 15283(0.13%) |
| E21-1 | 15234778 | 837168(5.50%) | 18093(0.12%) | 8953(0.06%) | 9679(0.06%) | 15224(0.10%) |
| E21-2 | 14992767 | 191815(1.28%) | 5611(0.04%) | 3378(0.02%) | 8175(0.05%) | 11614(0.08%) |
| E21-3 | 12082571 | 397863(3.29%) | 10851(0.09%) | 5899(0.05%) | 8279(0.07%) | 20776(0.17%) |

Table S2 Alignment non-coding RNA in Rfam

| sample | total | rRNA | snRNA | snoRNA | tRNA |
| --- | --- | --- | --- | --- | --- |
| all | 163303515 | 3082952(1.89%) | 112818(0.07%) | 137984(0.08%) | 373613(0.23%) |
| E12-1 | 14257768 | 453695(3.18%) | 19920(0.14%) | 16150(0.11%) | 43261(0.30%) |
| E12-2 | 14795551 | 437354(2.96%) | 18021(0.12%) | 17404(0.12%) | 40094(0.27%) |
| E12-3 | 12960780 | 286682(2.21%) | 16492(0.13%) | 13946(0.11%) | 31639(0.24%) |
| E16-1 | 12839200 | 119624(0.93%) | 4855(0.04%) | 7562(0.06%) | 25200(0.20%) |
| E16-2 | 11612348 | 217801(1.88%) | 7785(0.07%) | 9310(0.08%) | 24972(0.22%) |
| E16-3 | 15238234 | 220907(1.45%) | 8594(0.06%) | 14008(0.09%) | 26860(0.18%) |
| E19-1 | 13135207 | 254586(1.94%) | 7446(0.06%) | 8921(0.07%) | 27302(0.21%) |
| E19-2 | 14707612 | 231672(1.58%) | 5949(0.04%) | 12368(0.08%) | 31678(0.22%) |
| E19-3 | 11446699 | 142455(1.24%) | 4788(0.04%) | 9446(0.08%) | 23954(0.21%) |
| E21-1 | 15234778 | 396032(2.60%) | 9486(0.06%) | 10386(0.07%) | 27504(0.18%) |
| E21-2 | 14992767 | 118912(0.79%) | 3148(0.02%) | 8633(0.06%) | 19349(0.13%) |
| E21-3 | 12082571 | 203232(1.68%) | 6334(0.05%) | 9850(0.08%) | 51800(0.43%) |

Table S3 Alignment the repeat area

| sample | total | DNA | LINE | LTR | SINE | others |
| --- | --- | --- | --- | --- | --- | --- |
| all | 163303515 | 105878(0.06%) | 12744(0.01%) | 54938(0.03%) | 162(0.00%) | 7646857(4.68%) |
| E12-1 | 14257768 | 21449(0.15%) | 1930(0.01%) | 7736(0.05%) | 36(0.00%) | 1110910(7.79%) |
| E12-2 | 14795551 | 17117(0.12%) | 1953(0.01%) | 4999(0.03%) | 27(0.00%) | 1052942(7.12%) |
| E12-3 | 12960780 | 10081(0.08%) | 1360(0.01%) | 3931(0.03%) | 20(0.00%) | 590175(4.55%) |
| E16-1 | 12839200 | 11175(0.09%) | 1012(0.01%) | 5829(0.05%) | 14(0.00%) | 328386(2.56%) |
| E16-2 | 11612348 | 10938(0.09%) | 1035(0.01%) | 4998(0.04%) | 8(0.00%) | 459368(3.96%) |
| E16-3 | 15238234 | 9256(0.06%) | 1006(0.01%) | 4690(0.03%) | 9(0.00%) | 459970(3.02%) |
| E19-1 | 13135207 | 5182(0.04%) | 754(0.01%) | 3612(0.03%) | 12(0.00%) | 706712(5.38%) |
| E19-2 | 14707612 | 4942(0.03%) | 829(0.01%) | 4016(0.03%) | 9(0.00%) | 652715(4.44%) |
| E19-3 | 11446699 | 4085(0.04%) | 531(0.00%) | 3609(0.03%) | 12(0.00%) | 397824(3.48%) |
| E21-1 | 15234778 | 4974(0.03%) | 1226(0.01%) | 4509(0.03%) | 4(0.00%) | 1105308(7.26%) |
| E21-2 | 14992767 | 3656(0.02%) | 496(0.00%) | 1417(0.01%) | 4(0.00%) | 244834(1.63%) |
| E21-3 | 12082571 | 3023(0.03%) | 612(0.01%) | 5592(0.05%) | 7(0.00%) | 537713(4.45%) |

Table S4 Number of identified miRNA and tag abundance statistics

| sample | total | mirna num | tags uniq | tags total | ratio(%) |
| --- | --- | --- | --- | --- | --- |
| all | 163303515 | 721 | 4975 | 123000951 | 75.32% |
| E12-1 | 14257768 | 542 | 3110 | 9984074 | 70.03% |
| E12-2 | 14795551 | 529 | 3027 | 11141910 | 75.31% |
| E12-3 | 12960780 | 513 | 2945 | 10011724 | 77.25% |
| E16-1 | 12839200 | 470 | 2883 | 10138709 | 78.97% |
| E16-2 | 11612348 | 464 | 2785 | 9057056 | 78.00% |
| E16-3 | 15238234 | 469 | 2879 | 11892779 | 78.05% |
| E19-1 | 13135207 | 423 | 2619 | 9857529 | 75.05% |
| E19-2 | 14707612 | 447 | 2707 | 10947594 | 74.43% |
| E19-3 | 11446699 | 397 | 2467 | 8732308 | 76.29% |
| E21-1 | 15234778 | 410 | 2633 | 10808475 | 70.95% |
| E21-2 | 14992767 | 399 | 2540 | 11595721 | 77.34% |
| E21-3 | 12082571 | 404 | 2491 | 8833072 | 73.11% |

Table S5 Base-edited for each sample

| sample | total | mirna tag | ratio(%) |
| --- | --- | --- | --- |
| all | 163303515 | 12438292 | 7.62% |
| E12-1 | 14257768 | 1086969 | 7.62% |
| E12-2 | 14795551 | 864157 | 5.84% |
| E12-3 | 12960780 | 814795 | 6.29% |
| E16-1 | 12839200 | 929932 | 7.24% |
| E16-2 | 11612348 | 840855 | 7.24% |
| E16-3 | 15238234 | 1116772 | 7.33% |
| E19-1 | 13135207 | 957735 | 7.29% |
| E19-2 | 14707612 | 1342338 | 9.13% |
| E19-3 | 11446699 | 909483 | 7.95% |
| E21-1 | 15234778 | 1314978 | 8.63% |
| E21-2 | 14992767 | 1318388 | 8.79% |
| E21-3 | 12082571 | 941890 | 7.80% |

Table S6 Statistics for identifying the known miRNA

| sample | total | mirna num | tags uniq | tags total | ratio(%) |
| --- | --- | --- | --- | --- | --- |
| all | 163303515 | 1059 | 20654 | 12845504 | 7.87% |
| E12-1 | 14257768 | 477 | 6359 | 977085 | 6.85% |
| E12-2 | 14795551 | 441 | 6366 | 907707 | 6.13% |
| E12-3 | 12960780 | 381 | 5695 | 717123 | 5.53% |
| E16-1 | 12839200 | 531 | 6496 | 942984 | 7.34% |
| E16-2 | 11612348 | 387 | 5707 | 809130 | 6.97% |
| E16-3 | 15238234 | 379 | 6477 | 1225800 | 8.04% |
| E19-1 | 13135207 | 395 | 6066 | 1077369 | 8.20% |
| E19-2 | 14707612 | 351 | 6636 | 1246222 | 8.47% |
| E19-3 | 11446699 | 332 | 5228 | 1016173 | 8.88% |
| E21-1 | 15234778 | 535 | 6922 | 1216227 | 7.98% |
| E21-2 | 14992767 | 303 | 6122 | 1452758 | 9.69% |
| E21-3 | 12082571 | 319 | 5273 | 1256926 | 10.40% |

Table S7 Statistics of novel miRNA

| sample | total | mirna num | tags uniq | tags total | ratio(%) |
| --- | --- | --- | --- | --- | --- |
| all | 163303515 | 267 | 334 | 266267 | 0.16% |
| E12-1 | 14257768 | 204 | 253 | 31802 | 0.22% |
| E12-2 | 14795551 | 203 | 254 | 40632 | 0.27% |
| E12-3 | 12960780 | 215 | 249 | 34269 | 0.26% |
| E16-1 | 12839200 | 170 | 196 | 17033 | 0.13% |
| E16-2 | 11612348 | 171 | 204 | 20530 | 0.18% |
| E16-3 | 15238234 | 182 | 219 | 31803 | 0.21% |
| E19-1 | 13135207 | 118 | 165 | 15828 | 0.12% |
| E19-2 | 14707612 | 129 | 162 | 19920 | 0.14% |
| E19-3 | 11446699 | 104 | 135 | 14342 | 0.13% |
| E21-1 | 15234778 | 140 | 164 | 10498 | 0.07% |
| E21-2 | 14992767 | 103 | 140 | 14659 | 0.10% |
| E21-3 | 12082571 | 122 | 151 | 14951 | 0.12% |

Table S8 The primers for the RT-qPCR amplification

| Genes | Reverse transcription primer | Forward primer(5’-3’) |
| --- | --- | --- |
| gga-miR-130b-5p | GTCGTATCCAGTGCAGGGTCCGAGGTATTCGCACTGGATACGACAGTAGT | CGCCTCTTTCCCTGTTGC |
| gga-miR-363-5p | GTCGTATCCAGTGCAGGGTCCGAGGTATTCGCACTGGATACGACTCAAAA | CGGTGGATCACGATGCAA |
| gga-miR-30e-3p | GTCGTATCCAGTGCAGGGTCCGAGGTATTCGCACTGGATACGACGCTGTA | CGCGTTTCAGTCGGATGTT |
| gga-miR-10b-5p | GTCGTATCCAGTGCAGGGTCCGAGGTATTCGCACTGGATACGACACAAAT | CGCGTACCCTGTAGAACCGA |
| gga-miR-338-5p | GTCGTATCCAGTGCAGGGTCCGAGGTATTCGCACTGGATACGACACTCAG | GCGCGAACAATATCCTGGTG |
| gga-miR-499-5p | GTCGTATCCAGTGCAGGGTCCGAGGTATTCGCACTGGATACGACCTAAAC | CGCGCGTTAAGACTTGTAGTGAT |
| gga-miR-1729-5p | GTCGTATCCAGTGCAGGGTCCGAGGTATTCGCACTGGATACGACGACTAC | CGCGATCCCTTACTCACATGA |
| gga-miR-26a-5p | GTCGTATCCAGTGCAGGGTCCGAGGTATTCGCACTGGATACGACGCCTAT | GCGCGTTCAAGTAATCCAGG |
| U6 | CCATATTAGAAGCCCCTTTTTGT | F: TCGCTTCGGCAGCACATA  R: AATATGGAACGCTTCACGAA |
| PLCB1 | F: ACTTGAACTTGGTGGCGTTTC | R: CCAGAAATGCGTCCCTTGAC |
| SUCLG1 | F: TTTTACAGGCAAGCAGGGCA | R: ATAACCGTGGCAGAAGCACC |
| SFRP1 | F: CCGTCATGCAGTTCTTTGGC | R: CTGGAGACTTCGGTGGCATT |
| BRCA1 | F: TGGTGCTGCCTGGGTGAAAG | R: CTGCAAGGCAATGTGCTGGG |
| ACTB | F: CAGCCATCTTTCTTGGGTAT | R: CTGTGATCTCCTTCTGCATCC |
| Note: F: forward primer, R: reverse primer; miRNAs’ reverse primer(3’-5’) was consensus primer, primer sequence is AGTGCAGGGTCCGAGGTATT. | | |

Table S9 The predicted miRNA target genes

Based on the sequences of the existing miRNAs, known miRNAs and novel miRNAs, three softwares RNAhybrid(Version 2.1.2)+svm_light(Version 6.01), Miranda(Version 3.3a) and TargetScan(Version 7.0) were used to predict targets. The intersection of the results were more credible to be chosen as predicted miRNA target genes. At the same time, according to the predicted results, miRNAs with the same target gene are classified and listed in Table S9.

Figure legends

Figure S1. Sample tags length distribution. (1) E12-1; (2) E12-2; (3) E12-3; (4) E16-1; (5) E16-2; (6); E16-3; (7) E19-1; (8) E19-2; (9) E19-3; (10) E21-1; (11) E21-2; (12) E21-3.

Figure S2. Comparison of reference area statistics. Sample reference ratios distribution (1) E12-1; (2) E12-2; (3) E12-3; (4) E16-1; (5) E16-2; (6); E16-3; (7) E19-1; (8) E19-2; (9) E19-3; (10) E21-1; (11) E21-2; (12) E21-3. Replace different components ratios with different colors.

Figure S3. The first nucleotide bias distribution. Replace different nucleotides with different colors.

Figure S4. The first nucleotide bias with known miRNAs. Replace different nucleotides with different colors.

Figure S5. Tags annotation for all samples. (1) E12-1; (2) E12-2; (3) E12-3; (4) E16-1; (5) E16-2; (6); E16-3; (7) E19-1; (8) E19-2; (9) E19-3; (10) E21-1; (11) E21-2; (12) E21-3. Replace different components ratios with different colors.

Figure S1


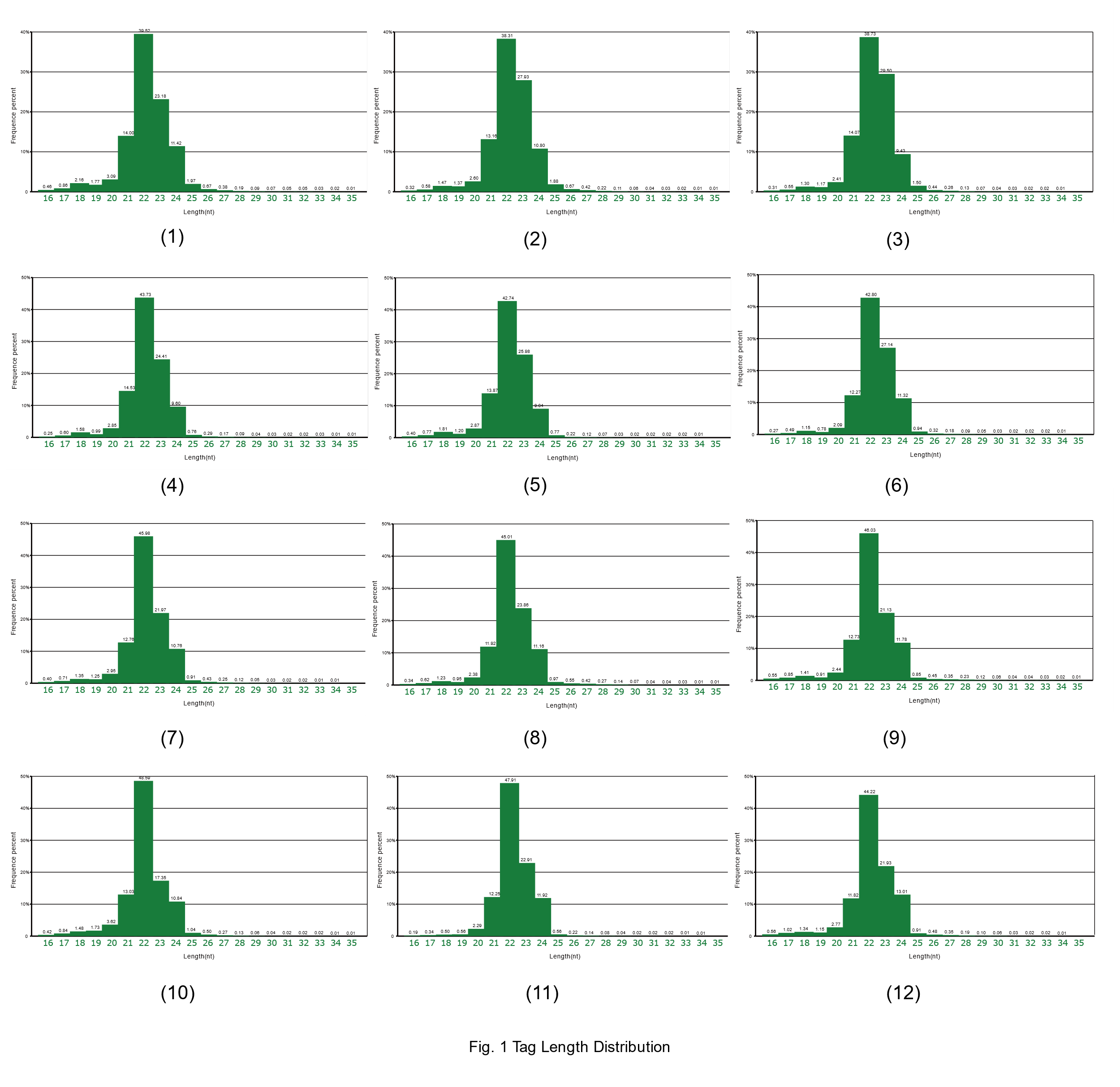


Figure S2


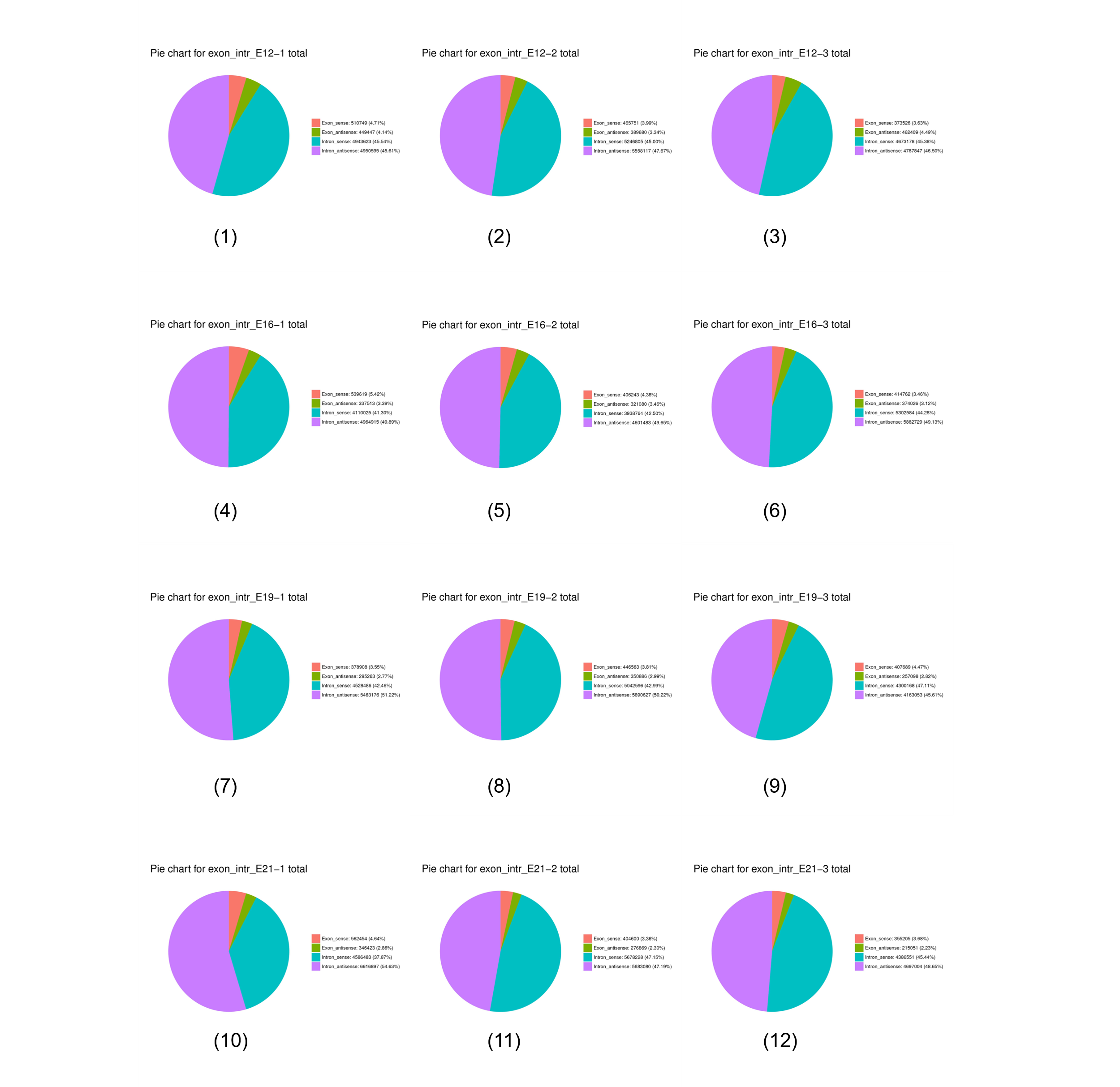


Figure S3


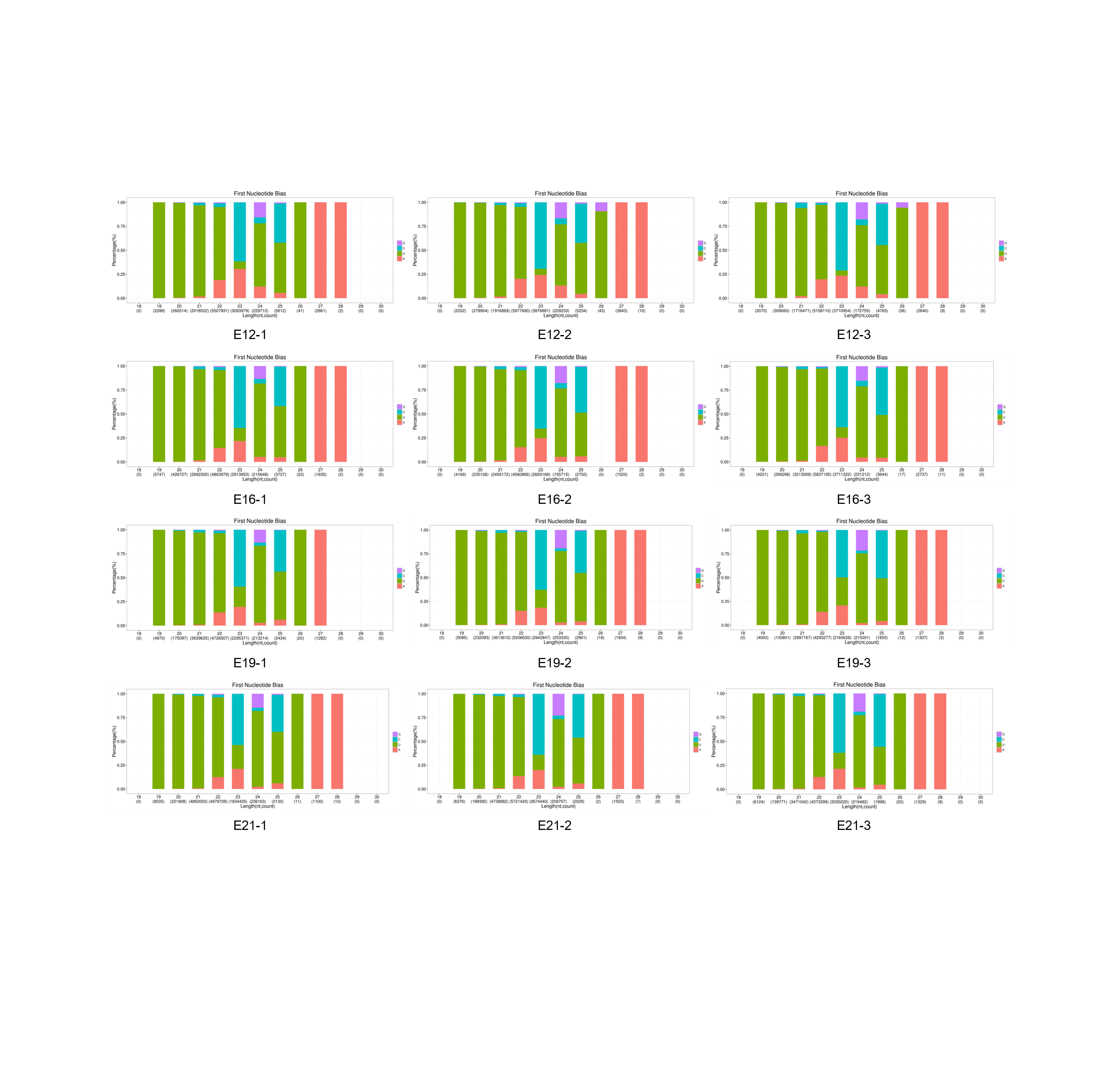


Figure S4


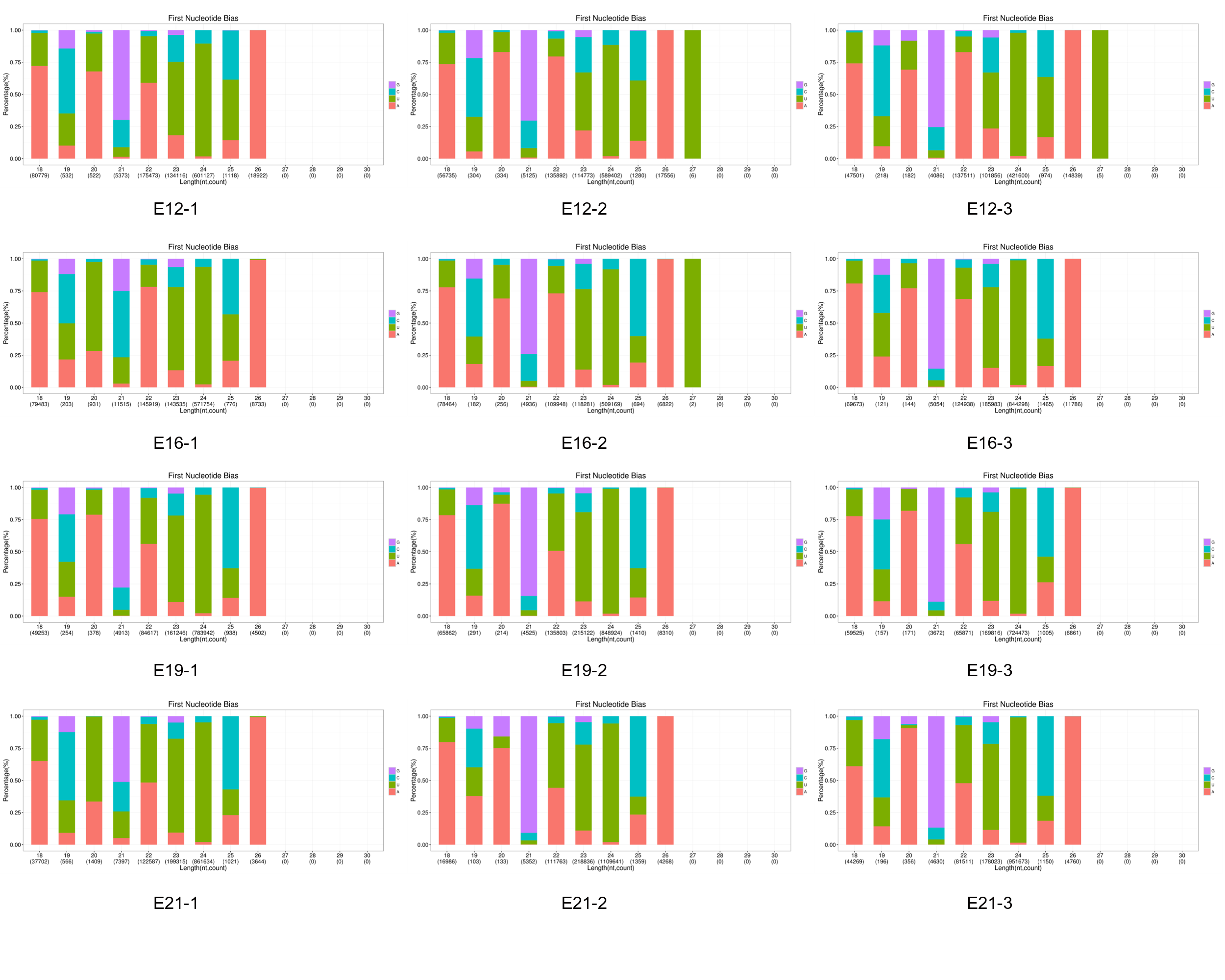


Figure S5


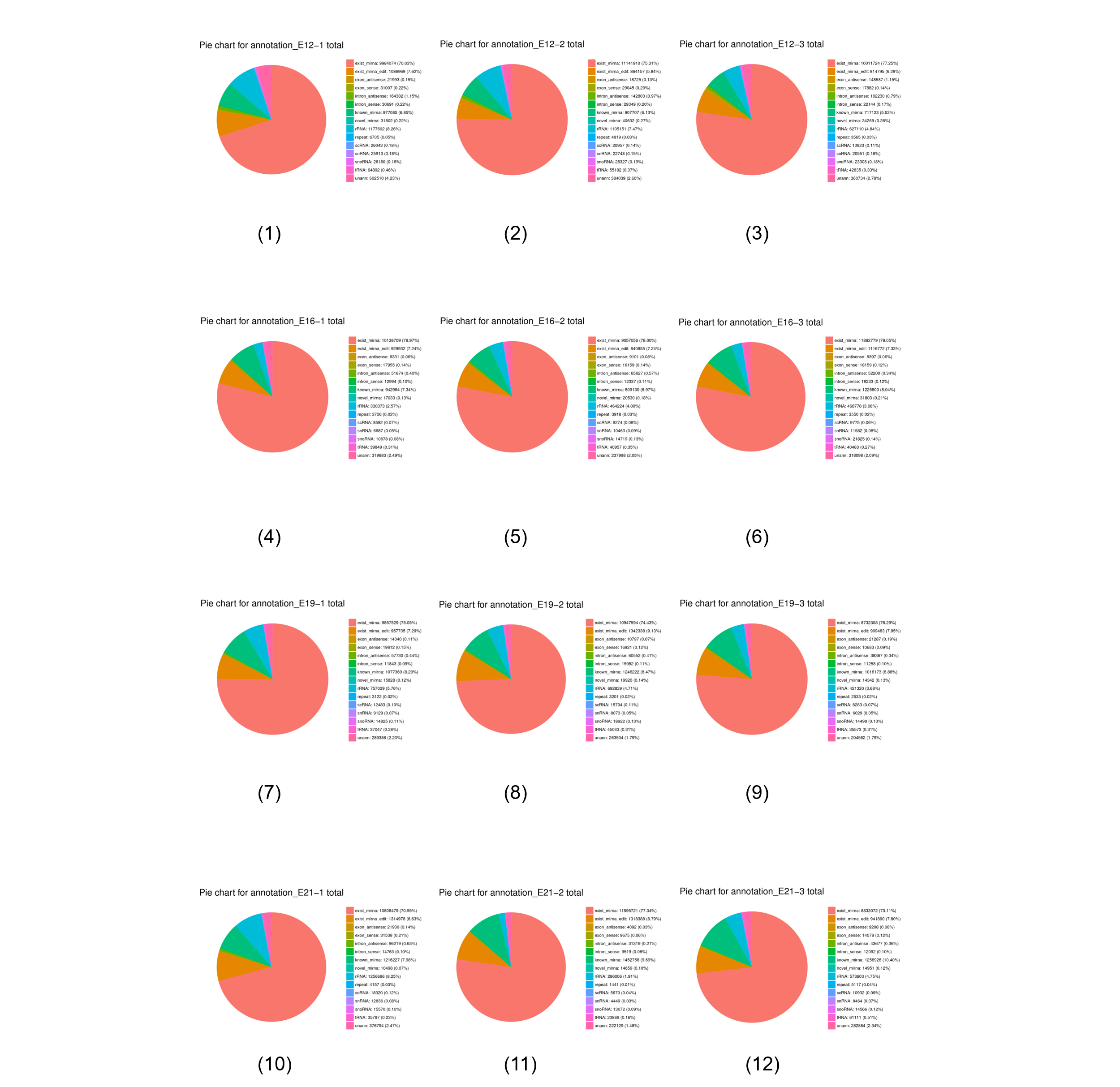

Supplement: Supplementary file 1 — Additional file 1: Table S1. Alignment non-coding RNA in GenBank. Table S2. Alignment non-coding RNA in Rfam. Table S3. Alignment the repeat area. Table S4. Number of identified miRNA and tag abundance statistics. Table S5. Base-edited for each sample. Table S6. Statistics for identifying the known miRNA. Table S7. Statistics of novel miRNA. Table S8. The primers for the RT-qPCR amplification. Figure S1. Sample tags length distribution. (1) E12-1; (2) E12-2; (3) E12-3; (4) E16-1; (5) E16-2; (6); E16-3; (7) E19-1; (8) E19-2; (9) E19-3; (10) E21-1; (11) E21-2; (12) E21-3. Figure S2. Comparison of reference area statistics. Sample reference ratios distribution (1) E12-1; (2) E12-2; (3) E12-3; (4) E16-1; (5) E16-2; (6); E16-3; (7) E19-1; (8) E19-2; (9) E19-3; (10) E21-1; (11) E21-2; (12) E21-3. Replace different components ratios with different colors. Figure S3. The first nucleotide bias distribution. Replace different nucleotides with different colors. Figure S4. The first nucleotide bias with known miRNAs. Replace different nucleotides with different colors. Figure S5. Tags annotation for all samples. (1) E12-1; (2) E12-2; (3) E12-3; (4) E16-1; (5) E16-2; (6); E16-3; (7) E19-1; (8) E19-2; (9) E19-3; (10) E21-1; (11) E21-2; (12) E21-3. Replace different components ratios with different colors. [file 12864_2022_8795_MOESM1_ESM.docx]
